# Abscess of Bone

**Published:** 1890-09-27

**Authors:** 


### The Practitioner's Mirror and Retrospect.

[*The Editor will be glad to receive offers of co-operation and contributions from members of the profession. There is no doubt that much valuable material full of interest and instruction is at present lost to science. This is largely so in the case of* (1) *the younger and less-known members of the hospital staff* (2) *those connected with cottage hospitals, and* (3) *the great body of medical practitioners not attached to any institution. The co-operation of all is cordially invited to enable the Editor to make* The Hospital *of the utmost possible use and value to the profession. Specialists willing to review books on their special subjects are invited to communicate this fact at once. All letters should be addressed to* The Editor, The Lodge, Porchester Square, London, W.]

### SURGERY.

### ABSCESS OF BONE.

A surgeon, writing more than 150 years ago, observed :—"That species of corruption of the bones, which takes its rise in their internal parts, and by degrees enlarges the bone, and raises it into a tumour, is at this time called by physicians and surgeons a *spina ventosa*, and by some *spinæ ventofitas*. . . . . This disorder seems to have borrowed the term *spina* from the resemblance which the eminences in the bone bear to thorns continually pricking the flesh and producing grievous pain, and the epithet *ventosa* is added because the tumour appears upon touching to be filled with wind or air, though in fact it is never, or very seldom, distended with air. . . . . . A spina ventosa is, therefore, understood to be a corruption of the bone occasioned by a depravity of the contained fluids and arising generally spontaneously without any external cause, beginning not upon the external face of the bone, but between its lamellæ or cells." Then this old surgeon, after detailing various methods of treatment by decoctions of woods, sarsaparilla, guaiacum, juniper, &c., says, if they fail abscess is to be feared, and advises laying the bone bare with the knife, and then making several small foramina in the bone with a small "piercer," and he gives a woodcut of the drill which he calls a piercer." Then the wound is to be dressed with "cleansing and balsamic applications" such as "Decoctum Agrimon, Saniculæ, Hyperici, vel Aristolochiæ, cum Melle, Rosar, aut Essent. Myrrhæ ac aloes; or a solution of Mercurius Dulcis in Aquâ Plantag. vel Aqua Calcis." The next author who appears to have written on the subject was William Bromfield, who published his "Chirurgical Observations" in 1773. In 1824 Sir B. Brodie, following the practice of the day, amputated a limb for incurable pain in the tibia, finding a cavity filled with pus. And in 1846 Sir B. Brodie published an article giving an account of chronic abscess of the bone, having previously trephined for the relief of the malady. Sir B. Brodie then expressed his opinion that trephining for bone abscess would be the means of preserving many limbs when it was more generally adopted.

Mr. Jordan Lloyd, in some recent observations in the *Provincial Medical Journal* on abscess of bone, remarks that "many surgeons are still unaware of the meaning of a group of symptoms diagnostic in his opinion of a collection of degenerating inflammatory products within the substance of a bone." Such cases are generally regarded as ostitis or periostitis. At an early stage this is undoubtedly correct. The symptoms of diagnostic significance on which Mr. Jordan Lloyd relies are localised paroxysmal pain, circumscribed exquisite tenderness, and chronicity. There may also be limited enlargement, increased local temperature, and wasting of the muscles of the limb. Attacks of pain, dull aching, and occasionally stabbing occur towards evening. The tenderness is limited probably to the size of a shilling, and has been described by sufferers as "like a boil in the bone." Local enlargement depends more on thickening of the periosteum than on expansion of the bone itself. The tibia is the bone most commonly affected, and it occurs most frequently in boys, and in men beyond forty years of age. Blows and syphilis are ordinary assignable causes, but it sometimes follows fevers—especially typhoid—and there is reason to believe it is often connected with the strumous diathesis. Mr. Jordan Lloyd relates several cases. A young man after a long walk felt sudden pain in the right internal malleolus. He was treated with splints and starched cases; also had a seton in front of the ankle joint, and for four months had used crutches. On coming under Mr. Lloyd's care there was paroxysmal localised pain in the right internal malleolus, with circumscribed tenderness and slight swelling, but no œdema or redness of the skin. The limb was sterilised by washing, emptied of blood by elevation, and a rubber tourniquet applied below the knee. A half-inch trephine was applied one inch above the tip of the malleolus, and a disc of bone three quarters of an inch in thickness removed. A cavity was found containing gummy pus and a barley-corn sequestrum. The cavity was scraped and the wound closed with silver sutures. Lister's dressings were applied and splints used. The wound was dressed on the fifth day and found to be healed. The patient has had no further trouble since. Mr. Jordan Lloyd remarks that the sudden onset after a long walk is interesting. What probably happened was that a small area of bone became congested, inflamed, and perished, a limited necrosis and abscess resulting. He remarks on the advantage of operating on a limb in a bloodless condition, as a small cavity can then be scarcely overlooked. Attention is also directed to immediate closure of the wound resulting in primary union, which the author considers preferable to packing cavities in bone for the purpose of healing them from the bottom by the long and painful process of granulation. Another case reported is that of a boy who complained of "tooth-ache in the leg." Another case was abscess in the upper end of the tibia after typhoid fever. Others occurring in persons with inherited syphilis also presented. In all a similar treatment was pursued, and satisfactory results obtained. In conclusion, Mr. Jordan Lloyd remarks that the majority of destructive joint diseases have their origin in neighbouring bones. So that as a prophylactic to joint disease, the early treatment of bone abscess is of great importance.

